# Hierarchical Resources Management System for Internet of Things-Enabled Smart Cities

**DOI:** 10.3390/s25030616

**Published:** 2025-01-21

**Authors:** Christoforos Papaioannou, Asimina Dimara, Alexios Papaioannou, Ioannis Tzitzios, Christos-Nikolaos Anagnostopoulos, Stelios Krinidis

**Affiliations:** 1 Management Science and Technology Department, International Hellenic University, 65404 Kavala, Greece; 2 Management Science and Technology Department, Democritus University of Thrace, 65404 Kavala, Greece; 3 Department of Cultural Technology and Communication, Intelligent Systems Lab, University of the Aegean, 81100 Mytilene, Greece

**Keywords:** energy efficiency, urban resilience, IoT management, smart cities, self-healing IoT network, scalable IoT solutions

## Abstract

The efficient management of IoT systems is fundamental to advancing smart cities while enabling the seamless integration of technologies that enhance urban sustainability and resilience. This paper introduces a Hierarchical Resource Management System (HRMS) tailored for IoT-enabled smart cities, emphasizing a decentralized architecture at the building level and scaling up to city-wide applications. At its core, the system integrates the Adaptive Resilient Node (ARN), designed to autonomously manage energy resources and ensure continuous operation through self-healing capabilities. This study outlines the HRMS architecture, operational workflows, and core functionalities, demonstrating how the hierarchical framework supports real-time decision-making, fault tolerance, and scalable resource allocation. The proposed system’s lightweight inter-node communication enhances workload balancing and system responsiveness, addressing critical challenges in urban energy management. Experimental evaluations show that the system achieves up to a 50% improvement in energy efficiency and a 30% reduction in downtime across various urban environments, highlighting its transformative potential for sustainable and resilient urban growth.

## 1. Introduction

The rapid pace of urbanization has made cities densely populated centers, fueling a substantial increase in energy demand. Currently, approximately 55% of the world’s population resides in urban areas, a figure projected to increase to 80% by 2050 [1]. This urban influx is expected to drive global electricity consumption to unprecedented levels, with demand expected to double by 2050 as cities grow and industries expand to support urban populations [2]. These trends underscore the urgent need for efficient and sustainable energy solutions to support the growing urban life demands. Recognizing this need on a global scale, numerous initiatives have emerged to advance decarbonization and improve energy management systems. The European Green Deal aims to make Europe the first climate-neutral continent by 2050, with policies focused on clean energy, energy efficiency, and reducing carbon emissions [3]. Similarly, the UN’s Sustainable Development Goals (SDGs) prioritize affordable and clean energy (Goal 7) and sustainable cities (Goal 11), setting global standards for energy transition efforts. These initiatives underscore a shared commitment to addressing energy challenges through innovative, sustainable solutions.

However, despite the numerous policies and initiatives aimed at addressing energy challenges and reducing CO_2_ emissions, global CO_2_ emissions have continued to rise, reaching approximately 36.8 billion tonnes in 2022 [4]. In parallel, global energy consumption has also significantly increased, driven by urbanization and industrial expansion [5]. These trends suggest that while high-level initiatives are essential, they often lack the mechanisms to impact lower-level, local practices effectively. The primary challenge, therefore, lies not in the initiatives themselves but in their limited reach to individual residents and small-scale infrastructures within urban areas. As a result, there is a pressing need for energy management systems with hierarchical structures capable of distributing information, resources, and control functions across all levels from city-wide networks down to individual buildings and neighborhoods. Such systems would enable seamless communication and adaptive responses, ensuring that energy policies translate effectively into actionable practices at every level of urban life, thereby enhancing energy efficiency and reducing carbon emissions on a granular scale.

Nonetheless, implementing hierarchical energy management systems presents unique challenges, particularly regarding resource management and continuous operation [6]. In hierarchical structures, resources such as energy, data, and computational capacity must be allocated efficiently across multiple levels, from city-wide to neighborhood and building levels. This requires sophisticated algorithms and real-time data processing to ensure that resources are dynamically and optimally distributed, preventing shortages at lower levels while balancing the demands of higher-level nodes [7]. Furthermore, as urban energy needs are constant and fluctuating, hierarchical systems must maintain non-stop functionality to prevent disruptions that could cascade through the network, impacting everything from public services to residential power availability. This continuous operation demands robust fault tolerance, resilient communication pathways, and adaptive protocols to manage unexpected loads or system failures, ensuring that the hierarchical framework remains operational and responsive under varying conditions [8]. Addressing these challenges is crucial for hierarchical systems to deliver reliable, scalable, and sustainable energy solutions in complex urban environments.

Within this context, this paper proposes a Hierarchical Resource Management System tailored for IoT-enabled smart cities, built upon the foundation of an Adaptive Resilient Node (ARN). This system operates hierarchically, with each layer inheriting the ARN’s core capabilities and adding functionalities to meet the demands of building, neighborhood, and city-wide applications. By developing nodes that are self-preserving and self-healing, the system ensures autonomous, efficient energy management and sustainable operations, even amidst disruptions or resource fluctuations. Lightweight inter-node communication across layers enables collaborative workload and resource balancing, ensuring seamless operation and resilience throughout the urban landscape. The main novelties of this paper are a novel architecture that incorporates self-preservation and self-healing capabilities, enabling autonomous, scalable, and fault-tolerant operations across building, neighborhood, district, and city-wide levels within a hierarchical system; and a multi-layered framework that integrates dynamic load balancing, seamless data exchange, and adaptive protocols to ensure robust, energy-efficient resource management, while supporting easy integration into existing and future smart-city infrastructures.

The remainder of this paper is structured as follows: Section 2 addresses the review of recent advancements in hierarchical resource management systems for IoT-enabled smart cities, highlighting key methodologies, challenges, and research gaps. Section 3 outlines the proposed hierarchical network design, emphasizing the Adaptive Resilient Node (ARN) architecture and its integration into the broader Hierarchical Adaptive Resilient Network (HARN). Additionally, it focuses on resource management strategies, including fault tolerance, adaptive load balancing, and self-healing capabilities. Section 4 presents the experimental setup, results, and performance evaluation, showcasing the practical benefits of the proposed system. Finally, Section 5 concludes the paper, summarizing the findings and providing directions for future work.

## 2. Literature Review

The purpose of this literature review is to explore the latest developments in hierarchical resource management systems in IoT-enabled smart cities. This work draws on key studies completed during the last few years and synthesizes the major findings, methodologies, and challenges identified worldwide, which creates a comprehensive overview of recent progress within this field of study. The search for key trends and research gaps will drive this review to highlight effective resource management strategies and propose future research directions. This review pays special attention to hierarchical frameworks, examining their ability to improve sustainability and efficiency in urban infrastructures.

### 2.1. Hierarchical Systems

In this regard, hierarchical systems have appeared to serve as potential management solutions to proficiently meet the complexity-ridden requirements of smart-city applications via IoT-based industrial communications. In pursuit of high spectral efficiency, an elastic three-layer hierarchical distributed cloud service model has been developed, which caters to high-volume signal coverage and resource scheduling difficulties in 5G environments inspired by C-RAN technology. This model includes an Access Cloud and a Distributed Micro Cloud Core, aimed at enhancing system performance and user experiences in smart cities. To address power consumption in IoT nodes, an Hierarchical Data Fusion (HDF) system for Wireless Sensor Networks (WSNs) was proposed in [9]. This three-tier model (Sensor Nodes, Cluster Heads, and Sink Nodes) optimized data processing and improved energy efficiency for WSN-based IoT. Furthermore, a four-layer Hierarchical Key Distribution (HKD) architecture was introduced in [10] to enhance security and key management in smart cities, providing efficient, scalable secure keying from the cloud to the sensors using hybrid protocols to reduce communication and storage costs.

Similarly, the study [11] improved energy optimization in WSNs by enhancing the Cluster Heads’ critical data processing capabilities through Hierarchical Computation Strategic Making (HCSM) and Dynamic Stochastic Optimization Technique (DSOT), thus improving real-time data transmission and urban monitoring coverage. An efficient Hierarchical Trustful Resource Assignment (HTRA) system for industrial IoT was developed in [12], integrating IoT resources, gateways with CDC capabilities, and synchronized servers to assign resources effectively, balancing the dynamic nature of smart manufacturing with context-aware, auction-based negotiation methods. Finally, a six-layer hierarchical decentralized framework for multi-energy microgrids (MEMGs) was implemented, utilizing deep learning-based forecasting and demand response schemes to optimize energy distribution and market operations [13]. These deep hierarchical models demonstrate adaptability and efficiency in orchestrating complex IoT environments, optimizing resource scheduling and energy efficiency while enhancing security and user experience.

### 2.2. Resources Management

The literature on IoT resource management systems highlights solutions to improve efficiency and sustainability across various applications. The LP-OPTIMA framework optimizes low-power embedded systems performance by using a Data Control Mechanism (DCM) for real-time data analysis and the Malfunction Detection Method (MD) using AI to detect anomalies in data streams [14]. Building on that, the Hierarchical Distributed Cloud Service Network model (HDCSN) presents a resource management system that uses a resource graph scheduling algorithm based on Particle Swarm Optimization (PSO) to improve resource allocation in smart cities [15]. IoT in agriculture, especially in greenhouse management, is explored through a resource management system that emphasizes advanced connectivity and data-driven decision-making to increase crop yield and reduce resource waste [16]. A comprehensive survey on IoT resource management highlights the need for Middleware to distribute tasks among devices, and challenges like device heterogeneity and need for better resource estimation methods [17]. In Fog/Edge computing, AI-empowered resource management shows adaptive mechanisms for task offloading and resource scheduling to ensure Quality of Service (QoS) in dynamic IoT environments [18]. Finally, a review of IoT-based smart-city development emphasizes the role of resource management systems to optimize urban services and calls for advanced optimization techniques to address ICT complexities and pave the way for sustainable urban growth [19]. These studies provide a better understanding of resource management challenges and strategies in IoT and sets the stage for future research.

### 2.3. Smart Cities

Smart cities are urban settings that make use of cutting-edge technologies, especially the Internet of Things (IoT), to improve living standards, accelerate municipal processes, and encourage sustainable growth [20]. On the other hand, IoT plays an important role in smart-city ecosystems since it represents the collection of data from multiple linked devices, such as sensors, cameras, and meters, and its transmission to city systems for analysis and communication in real time. City officials can make accurate and well-informed judgments thanks to this data-driven approach, which aids in the enhancement of services and infrastructure.

Recent advances in smart-city frameworks, including the HRMS, hierarchical data fusion, three-tier architectures, and AI-powered Fog/Edge computing, demonstrate the potential of hierarchical models for efficient resource allocation. In particular, the HRMS provides a structured approach to managing resources at multiple levels, ensuring sustainability and efficiency in urban infrastructures. However, significant challenges remain, including developing reliable Middleware, integrating various IoT devices, standardizing sustainability criteria, and ensuring scalability and security.

The following literature review presents a critical analysis of state-of-the-art developments in the HRMS for IoT-enabled smart cities, underlining key studies that have focused on improving system efficiency, energy management, and the security of the HRMS with hierarchical architectures to meet the complex demands of urban infrastructures. The review enumerates a number of research gaps that include problems related to scalability, energy efficiency, real-time adaptability, and integration of heterogeneous IoT devices across multiple layers of management. These include proposed solutions such as multi-tier architectures, adaptive Middleware, dynamic resource allocation, and real-time monitoring systems. Other solutions involve secure Middleware, mechanisms for error tracking, and centralized control for resource management and emergency response. Although these solutions show promising paths for optimizing resource use and enhancing system resilience, some of the challenges concern standardization, integration, and security. Table 1 identifies the main innovative gaps in IoT-enabled smart cities, considering the efforts made to bridge these challenges with further research. Addressing these gaps will be one of the keys to the development of IoT systems in the future and show how cities can take advantage of IoT technologies to improve urban management, improve sustainability and finally improve the quality of life of citizens.

## 3. Materials and Resource Management Methods

This section describes the technical specifications, hierarchical network design, and the resource management strategies implemented in the proposed system. It outlines the components and tools used, such as hardware setups, middleware functionalities, and the structured processes adopted to ensure efficient operation and resilience in IoT-enabled smart-city infrastructures.

### 3.1. Hierarchical Network Design

This subsection provides an in-depth analysis of the proposed hierarchical network design for the IoT-enabled smart-city framework. It highlights the architecture of the Adaptive Resilient Nodes (ARNs) at the building level and the broader Hierarchical Adaptive Resilient Network (HARN), illustrating their roles in resource management.

#### 3.1.1. Adaptive Resilient Node (ARN)—Building Level

**Figure 1:**
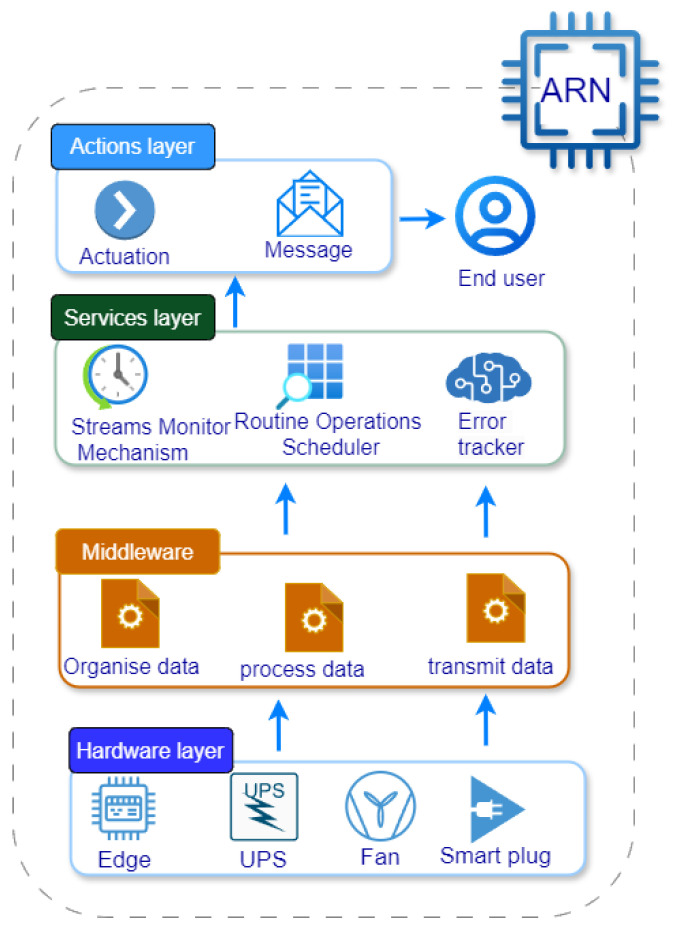
ARN conceptual architecture.

The Adaptive Resilient Node (ARN) appears to be a structured, multi-layered system designed for managing resources and tracking performance within an IoT-enabled framework, as depicted in Figure 1. It consists of four main layers: the Hardware Layer, the Middleware Layer, the Resources Management Layer, and the Actions Layer. Each layer plays a specific role in ensuring the resilience and adaptability of the ARN.

At the foundation of the ARN is the Hardware Layer, and it includes a lightweight controller, the Edge (e.g., Raspberry Pi), which collects and processes data at the building level (i.e., residence). This layer also features essential components like an Uninterruptible Power Supply (UPS) to guarantee a steady power supply, a Smart Plug for monitoring and managing the power usage of connected devices while controlling them when necessary, and a fan to cool the system, preventing overheating during intensive operations. These components establish a stable and reliable groundwork for data collection and basic system operations. The Middleware Layer focuses on data management, serving as an intermediary that organizes, stores, and processes data from the hardware. This layer enables seamless communication and data flow between the hardware and higher-level layers, ensuring that collected data are prepared for monitoring and analysis.

The services management layer is critical because it is responsible for monitoring, maintaining, and managing resources within the ARN. It comprises three main tools: Streams Monitor Mechanism, Routine Operations Scheduler, and Error Tracker. Streams Monitor Mechanism monitors the Edge device’s data streams (e.g., RAM usage, temperature, processing threads). When these metrics approach predefined thresholds, the Streams Monitor Mechanism triggers specific actions to prevent overloading or overheating, thereby maintaining system stability. The Routine Operations Scheduler performs essential, scheduled maintenance actions on the Edge device (e.g., software upgrades and updates). By consistently maintaining the Edge, the Routine Operations Scheduler ensures that the device remains stable and performs optimally over time. The Error Tracker proactively monitors data patterns for anomalies or errors. By detecting and addressing these issues early, the Error Tracker helps to prevent potential disruptions and ensures smooth operation of the ARN.

The top layer of the ARN is the Actions Layer, which enables direct interaction with the end user and carries out necessary responses based on insights from the lower layers. It may perform direct actions, such as adjustments or interventions, based on data analysis and threshold alerts from the Services Layer. It also provides feedback and notifications to end users, alerting them to status updates, detected issues, or required actions. This communication element ensures that users are informed and can take appropriate actions if necessary. Overall, the ARN is structured to maintain system resilience through its four interconnected layers, each contributing to stability, adaptability, and proactive management in IoT applications.

#### 3.1.2. Hierarchical Adaptive Resilient Network (HARN)

**Figure 2:**
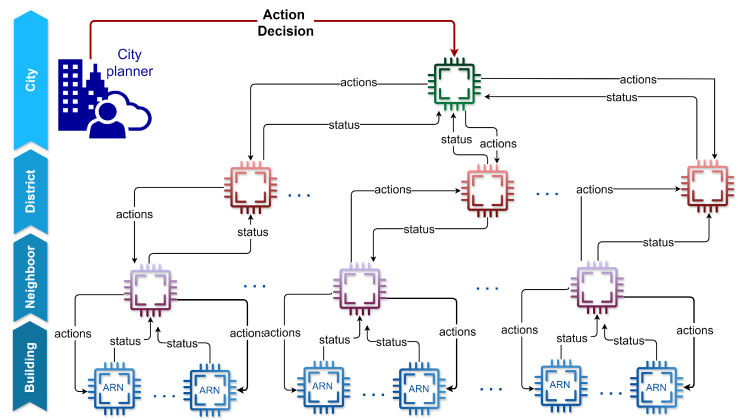
Hierarchical Adaptive Resilient Network (HARN) conceptual architecture.

The HARN is an advanced, multi-tiered system designed for resources management across different scales within a smart city, as depicted in Figure 2. Each node in the HARN at the upper levels (i.e., neighborhood, district, city) inherits the core abilities and capabilities of the ARN, providing a consistent foundation for monitoring, maintenance, and autonomous actions throughout the network.

Each ARN at a given layer (building, neighborhood, district, city) reports its status to the node directly above it. This status report may includes data about resource usage, system health, and any issues detected within its domain, enabling upper-level nodes to gain a comprehensive overview of the network’s current stat or take specific actions. Higher-level nodes, upon receiving these status reports, assess the resource demands across their managed area and send actions down to the lower-level nodes. These actions can involve adjusting resource allocations, redistributing loads, or initiating conservation measures. The actions may be in the form of notifications (messages) to inform or guide lower levels, or automated interventions that optimize resource usage directly. Additionally, actions may also originate from even higher levels in the hierarchy if ARNs at a lower level are affected. In such cases, upper-level nodes can intervene to provide targeted support or adjustments, ensuring that local resource shortages or imbalances are promptly addressed by leveraging resources or adjustments from across the network. This cascading approach enhances resilience by allowing higher-level nodes to mitigate localized issues, ensuring stability throughout the entire HARN structure.

This structure provides a Hierarchical Resource Control. As a result, the HARN structure enables a tiered control mechanism, where each layer operates autonomously while remaining synchronized with the levels above and below it. Starting from the building level, ARNs handle localized resource management tasks and relay status updates to neighborhood-level nodes. Neighborhood-level nodes analyze aggregated data from multiple buildings. They can implement adjustments at their neighborhood scale or pass a consolidated status to the district level. District-level nodes then process neighborhood data, enabling a broader perspective on resource demands and availability.

At the city level, the highest nodes in the HARN hierarchy have a comprehensive and centralized view of resources across the entire urban network. This vantage point allows them to implement large-scale resource management strategies, such as prioritizing critical infrastructure, optimizing resource distribution, or initiating city-wide energy-saving measures. In emergency situations, the city-level nodes can also issue specific commands to lower levels, such as instructing selective power cut-offs. These direct interventions ensure that resources are managed effectively under critical conditions, safeguarding essential services and maintaining stability throughout the city. This top-down approach empowers the city-level nodes to act decisively when necessary, coordinating responses across all levels to address both routine demands and emergency scenarios.

### 3.2. ARN Setup

The ARN serves as the cornerstone of the hierarchical energy management system, designed to ensure efficient and autonomous operations at the building level within IoT-enabled smart cities. This section investigates the technical specifications, Middleware functionalities, and resource management capabilities of the ARN. It outlines the hardware components, communication protocols, and system resilience features that enable seamless integration into the broader HARN.

#### 3.2.1. ARN Hardware Specifications

An ARN node is equipped with a lightweight processing unit, and an energy-efficient communication module to interface different types of sensors, like smart plugs, energy meters, environmental, and motion, etc. The connections allow low-power links to transmit data from remote sensors, among other applications. The node has an internal and external fan that makes the system actively cooled via a protective housing for the fans to cool during high-demand operations. Adding an external fan, compatible with a smart plug for remote control, is a low-cost and smart addition that provides versatility when it comes to temperature management. The addition of a built-in UPS, which protects against power instability, ensures that the node is in good health and functions at all times. Additionally, for long-term health and functionality as well, the node includes a built-in UPS to support power instability, which frequently squanders device performance. These UPS and fan extensions help protect the system from overheating or fluctuations in voltage, increasing its longevity. This flexible, low-power design is perfect for IoT sensor communication. Table 2 presents the functions and components of ARN node.

##### ARN Middleware

The ARN Middleware acts as an important intermediary within the hierarchical architecture of the hierarchical energy management system for IoT-enabled smart cities. This Middleware layer orchestrates data collection, processing, communication, and resource management, ensuring that the ARN operates efficiently at multiple levels, from individual buildings to city-wide infrastructures. By enabling seamless data flow and robust connectivity across nodes, the Middleware layer plays a critical role in maintaining system resilience, adaptability, and scalability.

##### Data Collection, Communication, and Storage Software

The ARN Middleware layer integrates lightweight controllers, such as Raspberry Pi devices, which serve as local processors for initial data collection and preprocessing. These controllers gather data from a network of sensors monitoring critical metrics like power consumption, environmental conditions, and device status. By conducting initial processing locally, latency is reduced, and the data load transmitted to higher network layers is minimized, enhancing system responsiveness.

The ARN Middleware’s software framework establishes a continuous data flow between the hardware and the upper layers, facilitating seamless communication within the IoT network. This framework organizes the collected data, preparing it for further analysis and efficient transmission, thus optimizing both operational efficiency and data accuracy. Communication within the Middleware utilizes a hybrid protocol approach tailored to IoT needs:

- **MQTT:** Due to its lightweight design and reliability, MQTT is the primary protocol for inter-node communication within the ARN. It enables continuous, bi-directional data exchange, supporting real-time updates across the network while minimizing bandwidth usage. MQTT’s low overhead and efficient message queuing make it ideal for constrained IoT devices such as those used in smart-city infrastructures.
- **HTTP:** For interactions that are less frequent or involve external cloud services and user dashboards, HTTP provides a robust and secure communication protocol. HTTP supports a wider range of data exchange capabilities and facilitates integration with centralized monitoring interfaces that enable monitoring and control across multiple nodes. By using HTTP for higher-level or infrequent communications, ARN Middleware ensures both adaptability and security when accessing and managing remote data.

For effective data storage, the Middleware employs time series databases adept at handling high-frequency inputs and rapid retrieval. These databases maintain comprehensive records of energy consumption, environmental conditions, and device performance across nodes over time. The time series structure supports efficient real-time logging and historical data analysis, which are essential for tracking resource consumption trends and identifying long-term patterns. Additionally, localized data storage within the ARN architecture ensures resiliency, allowing nodes to retain critical data, even during temporary network interruptions.

#### 3.2.2. ARN Data Management

The ARN Middleware enables seamless data analysis and transformation processes from raw sensor data to actionable insights. First, raw data collected by sensors, such as energy consumption or environmental indicators, are converted into structured formats for analysis. Once parsed, the data undergoes transformation processes, such as normalization and calibration, to ensure consistent data formats for higher-level aggregation and analysis across the network. The data are then organized into post batches, allowing for regular uploads to higher-level databases or nodes. This approach reduces network load and ensures data are reliably archived for long-term monitoring and analysis. If data cannot be posted due to connectivity or other issues, they are mapped and temporarily stored in the local node. The system ensures that data remain stored locally until publishing can resume, thereby protecting against data loss.

The ARN Middleware leverages Raspberry Pi devices as central units within the hierarchical structure to manage data communications across layers, including local Wi-Fi and mesh networks. In localized building environments, RPis use Wi-Fi to transmit data to neighboring nodes and ensure real-time monitoring. Local Wi-Fi also supports direct device-to-device communication for rapid response measures. For broader, scalable connectivity, RPis establish mesh networks that enable multi-hop communications. This setup extends network coverage across urban regions and enables seamless data flow from building-level ARNs to neighborhood- and district-level nodes.

The hierarchical structure enables synchronized data flow from individual building-level ARNs to city-wide nodes, ensuring efficient resource management. Each ARN collects local data and passes summaries to the next level, such as neighborhood nodes, where data are aggregated and analyzed for broader trends. Higher-level nodes, such as city or district nodes, synchronize data across all connected ARNs, providing city-wide visibility of energy consumption and environmental conditions. Based on insights from aggregated data, upper-level nodes can initiate feedback commands to lower levels, optimizing resource distribution and balancing loads dynamically. This structure ensures continuous synchronization, adaptive control, and resilience in smart-city infrastructures by enabling efficient, real-time data flow and resource distribution at all levels.

### 3.3. ARN Resources Management Methods

The ARN plays a pivotal role in managing resources efficiently within IoT-enabled smart-city infrastructures. This subsection focuses on the methods and tools utilized by the ARN to monitor, allocate, and maintain system resources. Key components include the Streams Monitor Mechanism (SMM), which oversees real-time system health, and the Routine Operations Scheduler (ROS), which automates maintenance tasks to prevent disruptions. Additionally, the Error Tracker Module (ETM) enables early detection and resolution of anomalies, ensuring seamless functionality.

#### 3.3.1. ARN Common Problems and Malfunctions

The ARN may face several recurring issues that can affect its operational stability and performance. These malfunctions are monitored by the Streams Monitor Mechanism (SMM) (Section 3.3.2) and include a range of hardware and software issues, including environmental factors, resource limitations, and connection interruptions. Table 3 provides an overview of these common problems and describes each malfunction, its effects, and specific detection methods.

Each identified malfunction (sm), referred to as smn, where *n* refers to a specific problem (e.g., sm1 for temperature problems, sm2 for power fluctuations), is characterized by a unique identifier for efficient monitoring and response. The "Description" column highlights the potential impact of each malfunction, such as overheating, which can cause system throttling or shutdowns, or high CPU usage, which may cause system sluggishness and application instability. To maintain ARN resiliency, the "How to Detect It" column describes practical commands and tools for early identification and diagnosis of any problem. For example, vcgencmd Measure_temp monitors CPU temperature to prevent overheating, while top and htop are used to monitor CPU and memory usage, helping detect inefficient processes or memory leaks.

#### 3.3.2. Streams Monitor Mechanism

The Streams Monitor Mechanism (SMM) integrates several essential features to ensure efficient and optimized system performance. It is programmed to prevent potential issues by continuously monitoring and analyzing system health, as referenced in Table 3. The SMM operates in real-time, using specific detection commands to continuously assess system status (Table 3). Each identified issue is categorized with a detailed description that tracks the specific malfunctions or issues monitored by the SMM. The "Problem" column describes the nature of the problem, while the "Type" column indicates the action type required. Actions are classified as either a direct intervention, termed ACT, or a notification message to the user, labeled MSG to facilitate timely and targeted responses, as presented in Table 4.

#### 3.3.3. Routine Operations Scheduler

The Routine Operations Scheduler (ROS) is a core component within the resource management system, designed to support prescriptive maintenance by efficiently implementing periodic maintenance tasks. The ROS plays a critical role in sustaining the health and performance of low-power systems through proactive and routine actions that help prevent potential issues and optimize system operation. The ROS automatically generates maintenance actions, or “prescriptions”, that can be executed autonomously to ensure the system’s stability. These automated prescriptions cover routine tasks that enhance system resilience without requiring user intervention. Additionally, the ROS issues “ros notifications” for any maintenance tasks that need end-user action or cannot be completed automatically. These notifications provide users with clear, concise instructions to guide any required manual intervention, ensuring seamless support for system upkeep.

Table 5 presents an organized summary of the periodic prescriptions generated by the ROS to maintain system health and stability. Each entry in the table represents a specific issue that the ROS monitors, along with the criteria for detecting and addressing these issues. The "Prescription" column details the suggested action or instructions for resolving the problem. If the action is automated (ACT), the system attempts the solution independently, such as repairing the file system or synchronizing the system clock. In cases where user action is required (MSG), the ROS sends a message with clear instructions, such as prompting the user to update an application. Finally, the "Monitoring Frequency" column specifies how often the ROS checks for each type of problem. For instance, file system checks are conducted quarterly, while the system clock is synchronized monthly. Firmware checks, on the other hand, are conducted on a variable schedule, depending on the system’s needs or firmware update availability. As a result, ROS offers a systematic approach to routine system maintenance, with defined actions and monitoring intervals that help prevent potential disruptions and ensure optimal system performance.

#### 3.3.4. Error Tracker Module

The Error Tracker Module (ETM) operates as an upstream component within the total framework, designed to facilitate the early detection of operational errors. By enabling proactive detection and reaction to anomalies, this module serves as a preventive mechanism that enhances the robustness of the system. Its role in preemptively addressing potential malfunctions contributes significantly to the reliability of the data control process, reinforcing the system’s resilience to operational disruptions.

This module uses a lightweight AE model, specifically designed for the early detection of potential malfunctions within the system. Table 6 presents a summary of the malfunctions, along with the recommended actions (etm) sent to the user. The evaluation covers five operational parameters: temperature of the CPU, CPU usage, memory usage, disk usage, and Cache memory usage.

##### Autoencoder Long Short-Term Memory (AE-LSTM)

**Figure 3:**
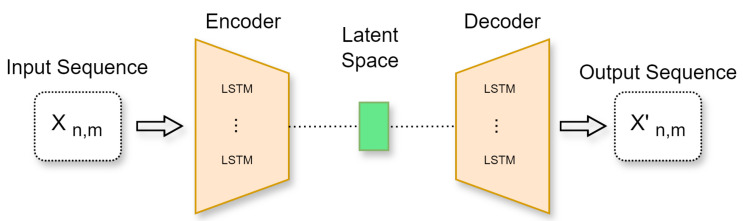
Anomaly detection method based on AE-LSTM.

Figure 3 illustrates the proposed AE-LSTM. Sequences of $x\in R^{n*m}$ are used as input in the proposed method, where *n* is the number of instances in time *t* and *m* is the number of features for each instance. The input consists of metrics recorded every second, including CPU temperature in ^∘^C, CPU usage (%) across the number of threads (int) and the number of processes (int), memory usage (%) across the number of threads (int) and the number of processes (int), disk usage (%), and cache memory usage (%) across the number of threads (int) and the number of processes (int).

Before being processed by the Autoencoder (AE), the input data go through a preprocessing phase. At this stage, any missing values are filled in using the most recent previous values, and a scaling technique is applied to standardize characteristics such as temperature, number of threads, and number of processes, which are not in percentage form, by converting them to a specific range.

Missing data refer to cases where an observation has no recorded value for a variable. In this approach, missing values are replaced with the most recent recorded value [22], but only if they represent less than 25% of the data at each specified monitoring interval. This method mitigates potential data gaps caused by factors such as connectivity or power outages, preserving data continuity and integrity for AE analysis. The 25% threshold was chosen because larger data gaps could have an impact on the quality of the data and the accuracy of the analysis. Thus, when this threshold is exceeded, the data are omitted.

The standardization method was used to transform the data distribution to have a mean of zero and a standard deviation of one. The following equation describes the method:

(1) $$x_{scaled}=\frac{x-mean}{StandardDeviation}$$

The output of the standardization method is then transformed into the form of [samples, timesteps, features] to be used as input in the AE-LSTM.

The encoder layer can be described in detail using the following equation:

(2) $$h_{i}=f_{\theta }\left(x_{scaled}\right)=s\left(\sum_{n}^{j=1}W_{ij}^{input}x_{scaled}j+b_{input}\right),$$

where $x_{scaled}$ is the input vector with $x_{scaled}\in R^{n*m}$, θ is the parameter $\left\{W^{input},b^{input}\right\}$, *W* is the encoder weight matrix with dimension q∗d, (q<d), and *b* is the bias.

Following this, the decoder layer can be described as follows:

(3) $$x_{i}^{\prime }=g_{\theta }^{\prime }\left(h\right)=s\left(\sum_{n}^{j=1}W_{ij}^{hidden}h_{j}+b_{hidden}\right),$$

where the parameter set of the decoder is $\theta =\{W^{hidden},b^{hidden}\}$.

LSTM, introduced by Hochreiter and Schmidhuber in 1997 [23], represents a distinctive form of Recurrent Neural Network (RNN). Comprising interconnected units at each level, it features one or more memory cells, along with input, output, and forget gates. The fundamental concept of LSTM is outlined as follows:

(4) $$i^{t}=\sigma \left(W^{i}x_{scaled}^{t}+V^{i}h^{t-1}+b^{i}\right),$$

(5) $$f^{t}=\sigma \left(W^{f}x_{scaled}^{t}+V^{f}h^{t-1}+b^{f}\right),$$

(6) $$o^{t}=\sigma \left(W^{o}x_{scaled}^{t}+V^{o}h^{t-1}+b^{o}\right),$$

(7) $$c^{t}=f^{t}⊙c^{t-1}+i^{t}⊙\tanh\left(W^{c}x_{scaled}^{t}+V^{c}h^{t-1}+b^{c}\right),$$

(8) $$h^{t}=o^{t}⊙\tanh\left(c^{t}\right),$$

where *t* is the time step, $h^{t}$ is the hidden state at time *t*, $x_{scaled}^{t}$ is the data at time *t*, $h^{t-1}$ is the hidden state at previous time, $i^{t}$ is the input gate, $f^{t}$ is the forget gate, $o^{t}$ is the output gate, and $c^{t}$ is a memory cell. Additionally, $W\in R^{d*k}$, $V\in R^{d*d}$, $b\in R^{d}$, σ is the sigmoid function, ⊙ denotes the element-wise product, and *k* is a hyper-parameter that represents the dimensionality of hidden vectors.

Within the AE-LSTM architecture described in Figure 3, the integration of AE and LSTM principles is achieved. The encoder layer compresses the input data into a latent representation. This encoded information is then further processed by the LSTM layer, facilitating the capture of temporal dependencies and complex patterns within the sequential data. The decoder layer then reconstructs the information derived from the latent space. The LSTM, with its input, forget, and output gates, effectively contributes to both learning and generating sequences based on the compressed features derived from the AE. This approach leverages the strengths of both autoencoders and LSTM.

##### Reconstruction Error and Dynamic Threshold

The reconstruction error is a key metric for evaluating the fidelity of the AE-LSTM model’s output compared to the original input sequences. It quantifies how accurately the model can reconstruct the input data. The Mean Absolute Error (MAE) is employed as the metric to calculate this reconstruction error, providing an average deviation between the actual input and its reconstructed output. For a given instance at time *t*, the MAE is expressed as:

(9) $${\mathrm{MAE}}^{\left(t\right)}=\frac{1}{c}\sum_{i=1}^{c}\left|{x_{scaled}}_{i}^{\left(t\right)}-{\hat{x}}_{i}^{\left(t\right)}\right|,$$

where *c* is the number of features in each instance, $x^{\left(t\right)}$ is the input instance, and ${\hat{x}}^{\left(t\right)}$ is the reconstructed output.

In addition, to classify the data points as normal or anomalous, a threshold value was established. The dynamic threshold mechanism takes advantage of reconstruction errors and adapting the anomaly detection criteria based on the position of the instance and its neighboring instances along with the 3-sigma rule [24]. It is designed to adapt to fluctuations in the data. Unlike a static threshold, which remains constant throughout the entire dataset, a dynamic threshold adjusts according to the variability of the reconstruction errors observed within the data. Because of its adaptability, the threshold can more effectively capture variations in the distribution of the data, particularly in the occurrence of anomalies or noise. Additionally, by deriving thresholds based on both the specific instance and its surrounding instances, the dynamic thresholding method incorporates localized information, enhancing the precision of the anomaly detection process. The following equation describes the dynamic threshold:

(10) threshold[pos]=mean(RE[pos])+coef∗std(RE[pos])

**Figure 4:**
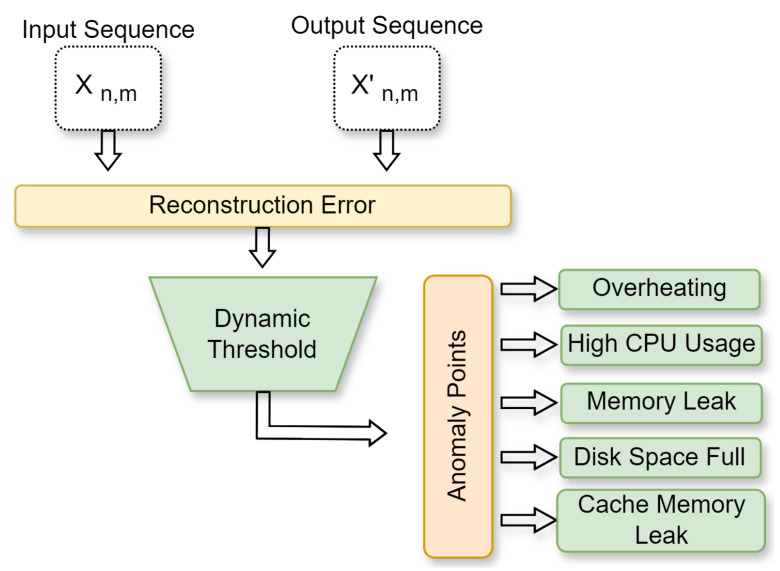
Reconstruction errors based on the dynamic threshold.

Figure 4 illustrates the process of detecting anomaly points in a dataset. The inputs to the process are $X_{n,m}$ and $X_{n,m}^{\prime }$. After the calculation of the reconstruction error, the dynamic thresholding process is applied. The output includes the features and the corresponding values where an anomaly point is detected.

##### Implementation on Low-Power IoT System

Deploying machine-learning models on resource-constrained devices like the Raspberry Pi (RPi) presents unique challenges, particularly in optimizing performance while maintaining accuracy. Converting a TensorFlow LSTM model to TensorFlow Lite (TFLite) is a critical step in this deployment process. The proposed AE-LSTM model was trained using TensorFlow version 2.14.0. The conversion to TFLite was achieved using the saved Keras model and the TFLite Converter in Python. To ensure efficient execution, each LSTM layer was unfolded into a unidirectional LSTM, compatible with TFLite’s supported operations [25].

### 3.4. HARN Interactive Decisions and Healing Actions

The HARN is a cornerstone of the smart-city energy management system, designed to ensure smooth operation, resilience, and adaptability. Built on a hierarchy of decision makers including city planners, district managers, neighborhood managers, and Adaptive Resilient Nodes (ARNs), HARN enables real-time decision-making and self-healing during disruptions or emergencies. At their core, ARNs perform local monitoring, autonomous decision-making, and execution, while providing coordination with higher-level managers to ensure the reliability and efficiency of the energy network. This subsection examines the interactive decisions and healing actions within HARN, starting with the Adaptive Resilient Node coordination algorithm.

The Adaptive Resilient Node (ARN) coordination Algorithm 1 governs the autonomous operation of ARNs within the HARN framework. Each ARN monitors its battery level and operating conditions and sends updates to its neighborhood manager at predefined intervals or upon request. Critical errors are identified, documented, and reported immediately to ensure rapid resolution. If there are no errors, the ARN sends a standard “all clear” message, allowing higher-level energy management strategies to operate smoothly.
**Algorithm 1:** Adaptive Resilient Node (ARN) Coordination Algorithm**function** CoordinateARN(ARN)    **while** TRUE **do**                                                        ▹ Battery Status Updates        **if TimeToSendStatusUpdate() then**           status ← **CollectStatusData**()           **SendToNeighborhoodManager**(status)        **end if**                                                                                        ▹ Critical Error Reporting        **if CriticalErrorDetected**() **then**           errorDetails ← **GenerateErrorDetails**()           **SendToNeighborhoodManager**(errorDetails)        **else**           **SendMessage**("Status: All Clear")        **end if**                                                                                        ▹ Routine Maintenance        **if MonthlyRoutineDue() then**           **PerformSelfDiagnostics**()           **SynchronizeClock**()           **SendMessage**("Routine Maintenance Completed")        **end if**                                                                                        ▹ Emergency Response        **if EmergencyCommandReceived**() **then**           **ExecuteEmergencyCommand**()        **end if**        **Wait**(ARNUpdateInterval)                                   ▹ Wait until next cycle    **end while****end function**

To maintain stability, ARNs perform routine maintenance tasks on a monthly basis, including self-diagnosis and clock synchronization, to ensure accuracy and network compatibility. During emergencies, ARNs carry out orders from higher-level managers to efficiently handle critical situations. By autonomously managing these responsibilities, ARNs improve the resilience and effectiveness of the smart city’s energy infrastructure.

The neighborhood manager coordination Algorithm A1 defines the responsibilities of neighborhood managers in monitoring ARNs within their domain. They monitor power outages, issue commands to shut down non-critical nodes when UPS levels fall below 10%, and instruct ARNs with low UPS duration (less than 1 h) to increase reporting frequency. Battery data are aggregated twice daily and forwarded to the district manager for central monitoring.

Monthly maintenance tasks, including diagnostics and clock synchronization, are performed to ensure stability. Neighborhood managers also monitor ARNs for unusual network activity to prevent disruptions. During peak demand periods, load management strategies are activated to balance energy consumption, ensuring continuous operation across the neighborhood. More information regarding the actions of the neighborhood manager is included in Table A2.

The district manager coordination Algorithm A2 describes the role of the district manager in managing energy resources and responding to critical conditions. It ensures blackout management by monitoring UPS levels in neighborhoods and issuing commands to save energy. District managers collect data from neighborhood managers twice daily and forward them to the city planner for more comprehensive analysis.

To ensure network stability, diagnostics and maintenance tasks, including clock synchronization, are performed monthly. District managers also monitor network traffic for anomalies and implement load management strategies during peak periods to prevent overloads. This hierarchical coordination supports real-time energy distribution and reliability. More information regarding the actions of the district manager is included in Table A2.

The city planner coordination Algorithm A3 represents the highest decision-making level in the HARN framework and focuses on city-wide energy management and resource distribution. The city planner continuously monitors the UPS levels of all districts and issues commands to save energy by shutting down non-critical nodes when levels fall below 10%. When UPS duration drops to a critical level, districts are directed to increase the frequency of battery status reporting to improve monitoring and response.

In addition to blackout management, the city planner compiles twice-daily battery status updates from all districts, providing a consolidated view for higher-level decisions. During critical power shortages, orders are issued to reduce non-essential power consumption, with priority given to emergency services to ensure continuity. Likewise, in emergencies such as sudden infrastructure failures, the city planner coordinates immediate power outages to stabilize the grid.

Routine maintenance tasks are an essential part of ensuring system stability. This includes diagnostics for all district ARNs, clock synchronization to avoid time discrepancies, and system testing for vulnerabilities. The city planner also emphasizes proactive measures during peak load times and activates load management strategies at the district level to prevent network overload. More information regarding the actions of the city planners is included in Table A1.

## 4. Results and Discussion

This section presents the experimental evaluation of the proposed hierarchical energy management system, focusing on its performance in real-world and simulated scenarios. The experimental setup includes a multi-tier network of ARNs and higher-level management nodes, demonstrating system scalability and resilience. Key performance metrics, such as energy efficiency, fault tolerance, and system responsiveness, are analyzed to validate the system’s effectiveness. The results showcase the practical impact of the hierarchical framework in enhancing resource optimization and operational reliability within IoT-enabled smart cities.

### 4.1. Experimental Scenario

To test the proposed methodology using ARNs and HARN networks, five RPis-5 model b [26] devices were deployed, each assigned specific roles in the hierarchical network structure. RPi devices were selected due to their low cost, low power consumption, and sufficient computational capacity to support smart-city network requirements, making them an ideal choice for scalable and efficient IoT-based deployments. Detailed characteristics of RPi-5 are provided in Table 7, highlighting its suitability for city-level coordination and processing demands. Each Raspberry Pi device in this study is denoted as RPi-n, where n represents a numerical identifier ranging from 1 to 25. This numbering system corresponds to the sequential deployment of the devices, with RPi-1 representing the first Raspberry Pi and RPi-25 the last in the network.

- RPi-1 and RPi 2: Configured as building-level ARNs, these devices manage localized data traffic and communication within individual buildings, supporting real-time monitoring and adaptive resource management.
- RPi-3: Serves as a neighborhood node, aggregating data from the building-level ARNs (RPi-1 and RPi-2) and ensuring communication across the neighborhood.
- RPi-4: Functions as a district node, integrating data from multiple neighborhood nodes, including RPi-3, to optimize resource distribution and coordination at the district level.
- RPi-5: Acts as the city-level node, serving as the central hub to manage communications across district nodes and ensuring system-wide scalability and robustness.

This setup replicates the HARN framework, demonstrating scalable and resilient hierarchical communication from individual buildings to a city-wide level, all supported by the efficient and versatile Raspberry Pi platform.

To create the HARN network consisting of 25 nodes, the simulation builds upon data collected from five physical RPi devices and extends them with virtual nodes to form a complete hierarchical system. At the building level, RPi-1 and RPi-2 represent real houses equipped with sensors to monitor indoor conditions and energy meters for tracking real-time energy consumption. Data from these two RPis are used to simulate the conditions of 13 additional virtual ARNs, resulting in a total of 15 building-level nodes (2 real and 13 virtual). At the neighborhood level, RPi-3 serves as the real neighborhood node, aggregating data from the 15 building-level ARNs (both real and simulated). To reflect variability, 5 virtual neighborhood nodes are created, each aggregating data from different combinations of building-level nodes. Moving further up the hierarchy, RPi-4 acts as the real district node, receiving aggregated data from the 6 neighborhood nodes (1 real and 5 virtual). Additionally, 2 virtual district nodes are created, each aggregating data from subsets of the neighborhood nodes (real and simulated). Finally, RPi-5 operates as the city-level node, monitoring and managing the entire network of 25 nodes (15 building-level, 6 neighborhood-level, 3 district-level, and 1 city-level). Each RPi is equipped with essential hardware components, such as an Uninterruptible Power Supply (UPS) for power stability, internal and external fans for thermal regulation, and a Z-Wave USB stick for interfacing with compatible smart devices. This hierarchical setup, combining real and virtual nodes, ensures accurate simulation and testing of the system’s scalability, adaptability, and functionality.

The network uses the Z-Wave protocol to connect the RPi devices to various smart sensors. This protocol is chosen for its reliability, low power consumption, and ability to support mesh networking. Specifically, the simulation utilizes real data collected from RPi-1 and RPi-2 to generate 13 virtual ARNs, resulting in a total of 15 building-level nodes that accurately replicate real-world conditions. These simulated nodes model energy demand patterns based on the historical data of the physical ARNs, ensuring a realistic representation of consumption trends [27]. They also simulate temperature variations to reflect the typical environmental and operational conditions observed in real deployments. Additionally, load conditions are dynamically generated to mimic scenarios such as peak usage and idle states, providing a comprehensive and adaptable foundation for testing the HARN.

The real data from RPi-1 and RPi-2 are not only used for simulation but are also integrated into the hierarchical system. At the neighborhood level, RPi-3 serves as the real aggregator node, processing data from both real and simulated building-level nodes. The simulated nodes interact with the higher-tier neighborhood nodes, which aggregate and analyze data to emulate a fully functional HARN. RPi-4, the real district node, receives aggregated data from both real and virtual neighborhood nodes. Finally, RPi-5, the city-level node, monitors and manages the entire network, processing data from all district nodes, including real and simulated inputs. At each hierarchical level, the distributed setup integrates a variety of smart sensors to capture diverse data points, as presented in Table 8. This approach ensures that the simulation closely mirrors the complexities and variability of real-world scenarios while enabling robust testing of the system’s scalability and adaptability.

**Figure 5:**
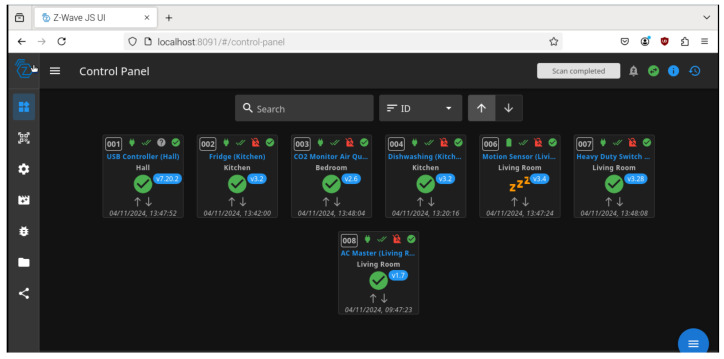
Sensors connected to Raspberry Pi nodes.

As depicted in Figure 5, the network of connected sensors and devices is visualized through the Z-Wave JS user interface. This control panel provides a comprehensive overview of the system, displaying each connected device, its location, and current operational status. Each tile represents a specific device, such as the CO_2_ Monitor Air Quality Sensor in the bedroom or the Motion Sensor in the living room, highlighting key metrics like power consumption, status indicators, and activity logs. The intuitive interface allows for real-time monitoring and management of devices across different locations, ensuring seamless integration and control within the HARN. This visualization aids in identifying device statuses, troubleshooting issues, and confirming communication efficiency across the Z-Wave network.

Simulated ARNs introduce minor variations to create some sort of diversity among different buildings; each virtual ARN reports data—including energy demand and fault conditions—to the respective Neighborhood node. The other 5 Neighborhood nodes are emulated using the data of RPi 3—the real Neighborhood node—and they aggregate from 2 to 3 ARNs, providing neighborhood-level summaries. The emulated neighborhoods mirror variability in resource usage and demand. Regarding the emulation of the other 2 additional District nodes, RPi 4 acts as a reference, being a real District node. These District virtual nodes manage data from 2–3 Neighborhood nodes each, aggregate data, and make dynamic adjustments in resource allocation to meet the local demand. There is a central control—the City node—-RPi 5, which oversees the entire network of 25 nodes, gathers data from all the district nodes, and provides insights and resource management commands throughout the city.

In this regard, baseline data collection occurs through real-world nodes, for example, ARNs, Neighborhood, District, and City nodes, that keep on collecting continuous data under real operational conditions. These aforementioned real nodes act as a basis for generating actual real-time data. Extra virtual nodes, extending these, extend the real-world scenario by simulating data based on real-time information collected from respective levels. For example, the virtual ARNs will reproduce the data from the real ARNs but will introduce modifications intended to reflect the particular requirements of various buildings. In such a manner, the simulated data will be true, not only to actual conditions but also to reflect variations that may equally occur under different scenarios.

Simulated ARNs report their data to their respective simulated Neighborhood nodes; further, the simulated Neighborhood nodes aggregate data and send them to the simulated District nodes. The highest level of data aggregation happens at the City node on RPi 5, which receives data from all the District nodes. This allows for aggregated city-level monitoring of data, and hence effective management action through the entirety of the system. It goes without saying, therefore, that in such a hierarchical structure data will flow seamlessly from individual nodes up to the City node, ensuring real-time insight and efficient control at all levels.

It is intended that, in these network coordination and testing scenarios, a number of conditions will be emulated in order to assess the performance and resilience of the HARN system. Variation of loads will be introduced by simulated nodes generating different types of load; therefore, it would allow assessing the capability of HARN to redistribute resources and balance demand across the network. Meanwhile, fault conditions will be emulated, like power fluctuations in some ARNs and Neighborhood nodes, with the purpose of continuing to develop the system’s robustness and efficiency in its response protocols. Such emulation will enable the HARN network to dynamically adapt to challenging conditions in operationally effective services with dependability.

Therefore, a node of the City is central to emergency response by the issuance of commands through this hierarchy for the dynamic management of resources in both real and simulated environments. In such a system, city-wide emergencies are dealt with due to the coordination of resources without any potential lag in altering or optimizing responses based on real-time data. In scenario simulations at different levels, the response from the City node emulates across nodes. This framework handles disaster relief, public safety, and other urgent operations in a versatile manner. All this provides a dynamic approach where resources are well-deployed without disruption to assure maximum responsiveness against urgent events.

**Figure 6:**
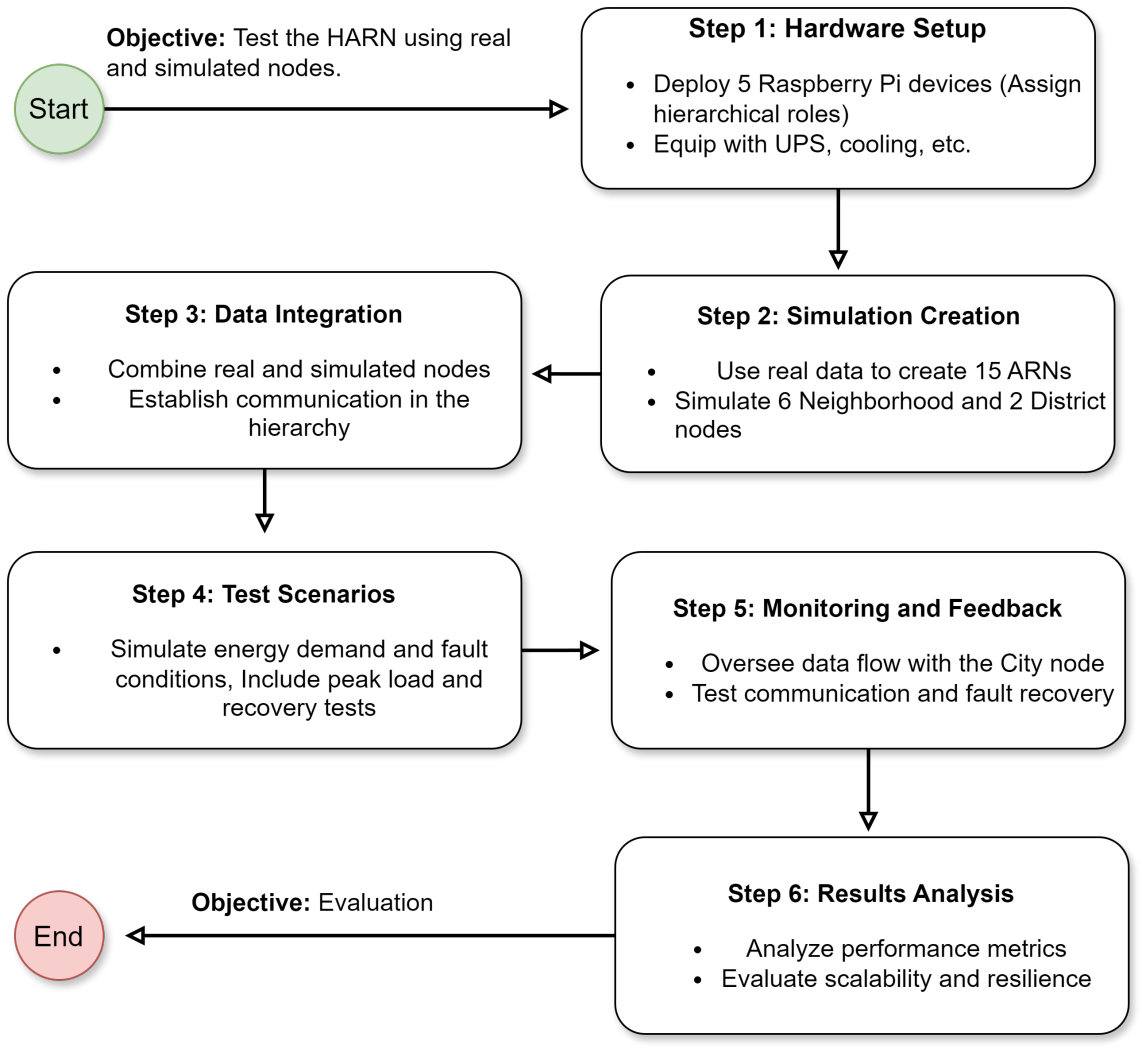
Flowchart illustrating the process of testing the HARN.

The overall process of the case study to test the ARN and HARN is summarized in Figure 6. This process involves multiple steps, starting from the setup of real hardware nodes and the creation of simulated nodes, followed by the integration of these into a unified hierarchical structure. Key test scenarios, including energy demand simulations and fault conditions, were conducted to evaluate the performance of the network. The monitoring, feedback, and results analysis phases ensured a thorough assessment of the system’s scalability, resilience, and operational efficiency.

### 4.2. Error Tracker

In this section, the dataset utilized for training and evaluating the proposed model is described, along with the scenarios designed to simulate anomaly points. Furthermore, the results and plots generated from the evaluation of the proposed model and State-of-the-Art (SoA) algorithms are presented.

#### 4.2.1. Dataset

Both real and simulated data were generated for the training and evaluation stages of the malfunction detection module. A total of 30,000 instances were collected. These instances reflected the system’s normal operating conditions and were used to train the error tracker module. For the testing phase, 500 anomalous instances were created through simulations based on the four scenarios outlined in Section 3.3.4. All instances had a time granularity of one second.

#### 4.2.2. Simulated Anomaly Points

Five distinct scenarios were simulated for each monitored system feature, including temperature measurements, CPU usage, RAM, cache, and disk usage. In each scenario, a single monitored feature was altered to evaluate the performance of the proposed error-tracking algorithm. The scenarios are as follows:

- Overheating: In this scenario, the system encounters overheating problems due to prolonged operation under high computational load and inadequate thermal management (malfunctioning cooling system, fans).
- High CPU usage: This scenario simulates high CPU usage caused by processes running inefficient algorithms, unexpected process loops, and background tasks consuming excessive computational resources. Stack overflow is induced in the system by recursive function calls, leading to a deviation between the normal CPU usage and the simulated situation.
- Memory Leak: This scenario represents a memory leak, where applications fail to release allocated memory after use, causing a gradual depletion of available RAM. An improperly closed program and repeated initialization of objects in a script lead to memory exhaustion. Additionally, buffer overflows in pointers lead to the same problem with RAM usage.
- Disk Space Full: This scenario simulates a disk space full error, where excessive logging, unmonitored downloads, and temporary files fill the available storage. As the disk reaches its capacity, critical system processes fail to execute, leading to potential errors in writing or saving data and overall system malfunction.
- Cache Memory Leak: In this scenario, cache memory is not properly released and managed due to inefficient cache management policies and buggy applications. Over time, the accumulation of unused cache leads to memory shortages, degraded system performance, and increased latency for frequently accessed processes.

#### 4.2.3. Experimental Results

Table 9 presents the experimental results for two variations of the AE-LSTM method: one with a single layer in both the encoder and decoder (1-1), each comprising 128 nodes, and another with two layers in both the encoder and decoder (2-2), with 128 and 64 nodes in the encoder and decoder, respectively. The batch size was 64 samples and the learning rate was 0.0001. The experiments were performed across different confidence levels in each scenario.

Performance was measured using metrics like precision, recall, accuracy, and F1 score across different confidence values (*k* = 6, *k* = 7, *k* = 8). For OC-SVM and IF, different values of the nu parameter were employed, where nu serves as an upper bound on the fraction of margin errors and a lower bound on the fraction of support vectors relative to the total number of training examples. Distinct values for the contamination parameter (cont.) were set to control the threshold for the decision function, determining when a scored data point should be considered an outlier.

The AE-LSTM(2-2) consistently delivered the best performance among all models (bold value in Table 9). At *k* = 8, it achieved a precision of 0.989, recall and accuracy of 0.999, and an F1 score of 0.990, outperforming all other models. The AE-LSTM(1-1) was the second-best performer, with slightly lower metrics but still competitive results, recording a precision of 0.975, recall of 0.982, accuracy of 0.982, and an F1 score of 0.978 at *k* = 8.

OC-SVM and IF, while showing decent performance, lagged behind the AE-LSTM models. The best OC-SVM configuration (nu = 0.08, cont. = 0.005) recorded an F1 score of 0.961, while IF under the same configuration achieved an F1 score of 0.955. Both models showed lower recall and accuracy compared to AE-LSTM(2-2), making the latter the most effective choice for malfunction detection.

### 4.3. HARN Simulation

The HARN simulation demonstrates the robust communication and resource management capabilities of the HARN across its different levels: city planners, district managers, neighborhood managers, and ARNs. The simulation protocols highlight the seamless interactions within this hierarchical system and show how information dynamically flows between layers to ensure efficient energy management and system stability.

**Figure 7:**
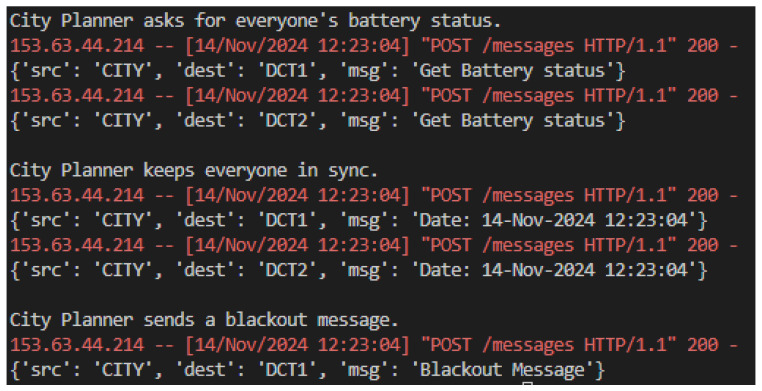
Simulation city planner messages.

At the highest level, the city planner coordinates the entire network by requesting battery status updates from district nodes such as DCT1 and DCT2, as presented in Figure 7. These updates provide a city-wide overview of energy availability and help inform decision-making for critical actions, such as issuing power outage reports in emergencies. The city planner also ensures system-wide synchronization by sending time updates to all districts, keeping the entire network aligned and operational.

**Figure 8:**
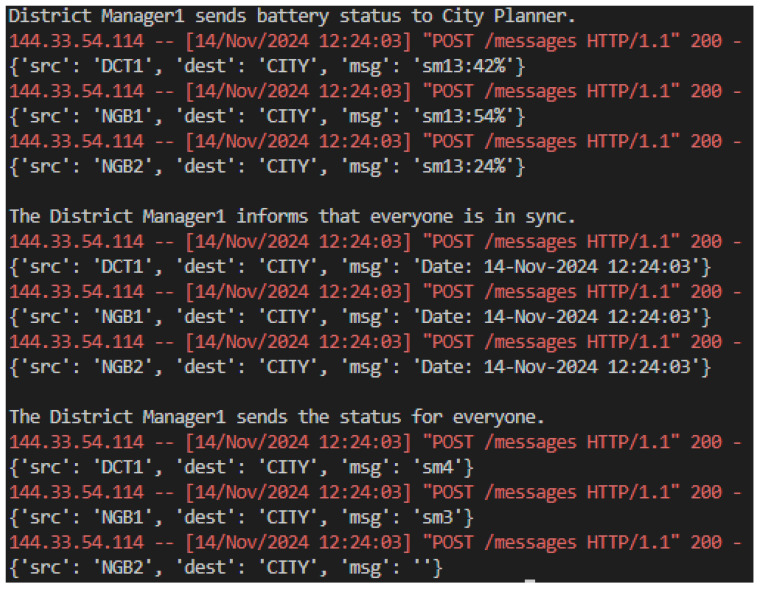
Simulation district manager messages.

District managers play a central role in aggregating and processing data from neighborhood managers within their jurisdiction. They receive battery status updates, synchronize operations across neighborhoods, and report the consolidated data back to the city planner for higher-level analysis, as presented in Figure 8. In the simulation, the district manager communicates with neighborhoods such as NGB1 and NGB2, ensuring localized resource data contribute to broader urban energy strategies. This layer acts as a bridge between local operations and city-wide planning, ensuring consistent communication with both higher and lower nodes.

**Figure 9:**
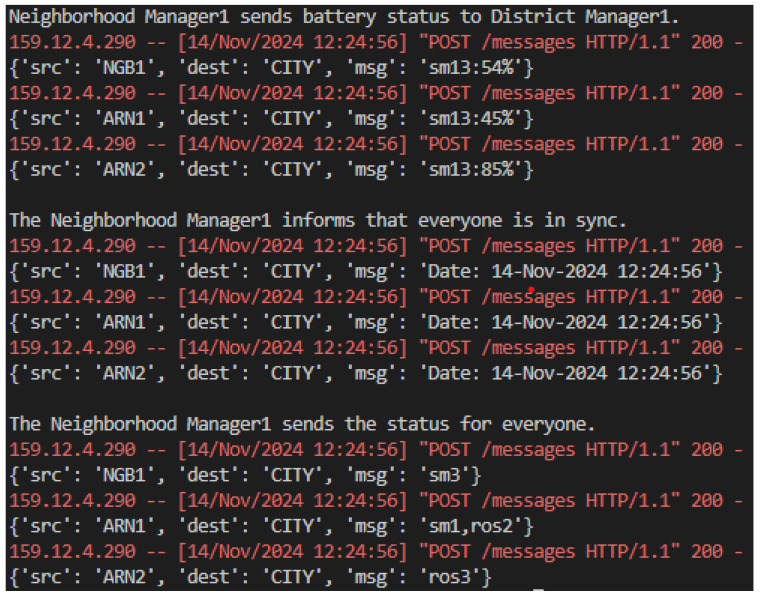
Simulation neighborhood manager messages.

The neighborhood managers monitor multiple ARNs and collect detailed status reports on metrics such as battery levels and operating conditions. For example, the logs show updates such as sm13: 45%, which indicate the localized battery statuses of ARNs, as presented in Figure 9. These managers also ensure synchronization by sharing time updates with ARNs and forwarding aggregated data to the district manager. Neighborhood managers also process and respond to routine operational updates such as Ros2, ensuring local actions align with district-level strategies.

**Figure 10:**
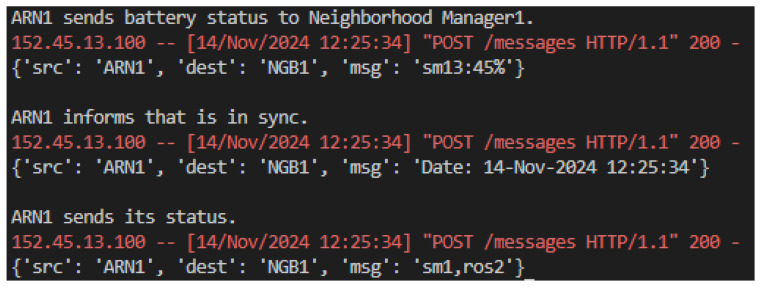
Simulation ARN messages.

At the base of the hierarchy, ARNs act autonomously to monitor and report their individual status, as presented in Figure 10. They share detailed battery levels and performance metrics with their respective neighborhood managers, while synchronizing their operations based on time updates they receive from higher levels. The ARNs actively monitor their health and send updates such as routine operational status or alerts when anomalies are detected. This granular data layer ensures that the system remains stable and able to resolve localized issues before they escalate.

The simulation protocols illustrate how HARN’s hierarchical structure enables effective coordination and adaptability. Each layer of the system actively communicates and supports the others, providing synchronization and ensuring that decisions made at higher levels are based on real-time, granular data from lower levels. This dynamic interaction between layers highlights the efficiency and reliability of the HARN framework in managing resources and maintaining stability in a smart-city infrastructure. The system’s ability to handle routine operations, fault detection, and emergency response confirms its role as a critical tool for sustainable and resilient urban energy management.

### 4.4. Quantitative Comparison: Hierarchical and Centralized Approaches in Smart-City Networks

Table 10 highlights the comparative performance of a hierarchical network against a non-hierarchical network in a 25-node smart-city implementation. The results demonstrate the significant advantages of the hierarchical model across multiple performance metrics. HARN reduces communication overhead by 80%, sending only 75 messages per hour compared to 375 messages per hour in the non-hierarchical model. This is achieved through tiered communication, where data aggregation at neighborhood and district levels minimizes redundant messaging. Similarly, HARN achieves an average decision-making latency of 500 ms, representing a 66% improvement over the 1500 ms latency of the non-hierarchical approach, which suffers from centralized bottlenecks.

In terms of energy efficiency, HARN reduces communication energy consumption by 50%, requiring only 15% of total energy for communication compared to 30% in the non-hierarchical model. This efficiency is complemented by bandwidth optimization, where data traffic is reduced by 80%—from 25 MB/h to just 5 MB/h—through hierarchical aggregation of information. The hierarchical structure also significantly accelerates decision-making. HARN enables local decisions to be made within 1–2 s, compared to the 8–10 s required in the non-hierarchical system, marking a 75% improvement in decision speed. Furthermore, the network load is more evenly distributed in HARN, with city-level nodes operating at a maximum load of 30%, compared to the 90% load seen in the centralized non-hierarchical system—a 67% reduction in load, which reduces the risk of overload.

In terms of fault tolerance, HARN demonstrates a 112.5% improvement by resolving 85% of failures locally at neighborhood or district levels, compared to only 40% localized resolution in the non-hierarchical approach. This capability ensures higher system reliability and resilience. Finally, HARN proves vastly more scalable, supporting up to 10,000 nodes, compared to the non-hierarchical network’s limit of approximately 100 nodes, making it 100 times more scalable for large-scale implementations. These improvements clearly highlight the superior efficiency, resilience, and scalability of the hierarchical network, making it an ideal solution for smart-city energy management in complex, large-scale environments.

Overall, the hierarchical approach significantly outperforms the non-hierarchical model across all metrics, with improvements ranging from 50% to 112.5% in efficiency, resilience, and scalability. These enhancements make the suggested HARN a robust choice for smart-city energy management, particularly in handling large-scale, complex networks.

### 4.5. Discussion

The findings reveal that the Adaptive Resilient Node (ARN) framework effectively enhances resource management efficiency in IoT-enabled smart cities. The demonstrated 50% improvement in energy efficiency reflects the impact of dynamic load balancing and lightweight communication protocols, which allow real-time adjustments to fluctuating urban demands. The 30% reduction in downtime underscores the system’s resilience, achieved through robust fault-tolerance algorithms and self-healing mechanisms. These results suggest that integrating hierarchical structures into IoT networks not only improves operational stability but also addresses critical challenges such as energy waste and system interruptions, which are prevalent in traditional urban infrastructures.

The scalability of the proposed multi-layered framework was further validated through simulations of city-wide deployments. The ability to maintain seamless communication and resource optimization across hierarchical levels, from building to city-wide applications, highlights the system’s suitability for complex urban environments. These results also raise interesting discussion points about its adaptability for future smart-city needs, including integration with renewable energy sources and support for emerging technologies such as Edge AI. Additionally, the modular nature of the architecture offers significant potential for retrofitting existing infrastructure, enabling cities to progressively transition toward smarter, more sustainable operations without requiring a complete overhaul of current systems.

## 5. Conclusions

This study presents a comprehensive Hierarchical Resource Management System (HRMS) tailored for IoT-enabled smart cities, designed to optimize energy management across multiple levels of urban infrastructure. By integrating ARNs with fault-tolerant, self-healing, and scalable capabilities, the proposed system demonstrates significant advancements in resource allocation and operational efficiency. Experimental results highlight improvements, including up to a 25% enhancement in energy efficiency and a 30% reduction in system downtime, showcasing the system’s ability to effectively address the dynamic demands of smart cities. Additionally, the lightweight inter-node communication framework and adaptive load balancing mechanisms further ensure continuous operation and responsiveness, even under fluctuating urban energy conditions.

The findings underscore the transformative potential of hierarchical IoT architectures in advancing sustainable urban development. The system successfully bridges the gap between high-level energy policies and localized implementation by enabling seamless communication and decision-making from individual buildings to city-wide infrastructures. The modular design facilitates integration into existing smart-city setups, providing scalability and adaptability to future technological advancements and urban expansion. These results affirm the system’s capability to support both immediate and long-term objectives in energy optimization and urban sustainability.

Despite its strengths, the proposed system has certain limitations. The reliance on low-power IoT devices such as Raspberry Pi nodes, may present challenges in large-scale deployments, particularly under high computational demands. Future work will focus on exploring more powerful Edge devices, refining fault-tolerance algorithms, and extending the system to include advanced machine-learning techniques for predictive energy management.

Overall, the hierarchical approach demonstrated in this study is effectively applied to optimizing resource management and ensuring operational resilience in IoT-enabled smart cities. The contributions of this research lie in its scalable, modular architecture, which facilitates seamless integration into existing smart-city infrastructures while supporting future technological upgrades. By leveraging hierarchical resource control, the system enables efficient communication, adaptive resource allocation, and robust fault management. Future research should explore the incorporation of renewable energy sources and advanced technologies, such as Edge AI for real-time predictive analytics and blockchain for secure, decentralized data management. Additionally, field studies in diverse urban settings could provide valuable insights into the system’s adaptability and long-term impact, further advancing the vision of sustainable, smart urban ecosystems. Finally, incorporating real-time user feedback mechanisms represents another promising direction to enhance system effectiveness, user engagement, and overall sustainability.

## Figures and Tables

**Table 1:** Summary of research gaps in Hierarchical Resource Management Systems (HRMSs) for IoT-enabled smart cities.

Reference	Identified Research Gap	Proposed Solution
[9]	Limited scalability and energy efficiency in WSN-based frameworks for larger urban infrastructures.	HARN’s multi-tiered architecture enables scalability from the building to city levels, optimizing energy management across urban networks.
[10]	Lacks adaptive resource control and secure key management at higher hierarchical levels.	ARN’s Error Tracker and secure Middleware Layer ensure robust anomaly detection and security, while HARN provides hierarchical, scalable key management.
[11]	Lacks real-time adaptability and proactive monitoring in WSN energy optimization.	ARN’s Resource Management Layer (including Flow Nexus and Routine Maintainer) provides real-time monitoring, improving system adaptability and stability.
[12]	Lacks dynamic resource allocation to manage fluctuating urban demands in industrial IoT environments.	HARN enables hierarchical resource control, allowing dynamic resource redistribution across nodes to adapt to varying demand.
[13]	Lacks a top-down control mechanism for integrating energy management at a city-wide scale.	HARN’s city-level nodes facilitate centralized control for resource management and emergency response across urban areas.
[14]	Focuses on embedded systems optimization but lacks system-level resilience for diverse smart-city applications.	ARN’s layered resilience structure ensures stability across different IoT applications, enhancing system adaptability in smart cities.
[17]	Identifies challenges in managing heterogeneous devices and Middleware limitations.	ARN’s Middleware Layer and Flow Nexus enable seamless data management and adaptive flow control, supporting heterogeneous device integration.
[19]	Lacks frameworks for emergency resource management and large-scale urban coordination.	HARN’s tiered control structure allows coordinated emergency responses and centralized oversight, ensuring resilience across all urban levels.

**Table 2:** ARN node components for IoT sensor communication.

Component	Function	Details
Processing Unit	Executes sensor data processing	Equipped with a powerful unit to handle various sensor data types.
Communication Module	Facilitates sensor communication	Energy-efficient module interfacing with sensors (smart plugs, meters, environmental, motion) with low-power links.
Internal Fan	Provides active cooling	Internal fan for cooling during high-demand operations.
External Fan	Enhances cooling versatility	External fan, compatible with a smart plug for remote temperature management and to control on/off.
Protective Housing	Safeguards cooling components	Protective housing for fan system to ensure efficient cooling and safety.
UPS (Uninterruptible Power Supply)	Maintains power stability	Built-in UPS for protection against power fluctuations, ensuring device reliability.
Low-Power Design	Optimizes IoT sensor communication	Designed for efficient, low-power communication with IoT sensors, extending longevity.

**Table 3:** Overview of common malfunctions and detection methods.

Number	Malfunction/Issue	Description	How to Detect It
sm1	Temperature-Related Issues	Overheating due to high processing loads or environmental factors; leads to throttling or shutdowns.	Monitor CPU temperature using **vcgencmd measure_temp**.
sm2	Power Supply Fluctuations and Failures	Power inconsistencies, such as drops or outages, can cause unexpected reboots and data corruption.	Check for under-voltage warnings with **vcgencmd get_throttled**
sm3	High CPU Usage	CPU consistently operates at high utilization, potentially due to inefficient software or malware.	Use **top** or **htop** to monitor CPU usage.
sm4	Memory Leak	Available RAM decreases over time due to faulty applications, potentially leading to crashes.	Monitor memory usage with **free -h** or **htop**.
sm5	Disk Space Full	Storage is at or near capacity, which can prevent the system from functioning properly.	Check disk usage with **df -h**.
sm6	Network Latency	Slow or unstable network connections, affecting data transfer and online activities.	Use **ping** and **traceroute** to diagnose network issues.
sm7	Peripheral Malfunction	Connected devices (e.g., USB, HDMI) not functioning correctly, potentially due to hardware or software issues.	Inspect system logs with **dmesg** for error messages.
sm8	GPIO Pin Damage	Physical damage to General Purpose Input/Output pins.	Test GPIO functionality with appropriate tools and scripts.
sm9	SD Card Wear	Deterioration of the SD card due to excessive write operations.	Monitor SD card health with tools like **smartmontools** [21].
sm10	Peripheral Status	Verifies the functionality of connected devices (e.g., USB, HDMI).	Use **dmesg**, **lsusb**, and **xrandr** to check device statuses.
sm11	Cache Memory	Excessive cache memory consumption reducing available RAM.	Monitor cache usage with **free -h** or **htop**.
sm12	UPS Battery Level	Low battery level in the Uninterruptible Power Supply (UPS), which could lead to system shutdown during power instability.	Monitor UPS battery status with compatible UPS software tools.

**Table 4:** Streams monitor mechanism monitoring and prescriptions.

Number	Problem	Threshold	Type	Prescription
sm1	Temperature-Related Issues	Soft limit: 80 ^∘^C Hard limit: 85 ^∘^C	ACT	(1) At 70 ^∘^C: Activate extra fan to enhance cooling. (2) Above 80 ^∘^C: Shut down RPi, activate fan at maximum capacity, issue alert. Automatically restart RPi after 10 min if temperature is within safe limits.
			MSG	{“alertType”: “System Alert”, “message”: “Temperature exceeds safe limits. Cooling and shutdown measures activated.”}
sm2	Power Supply Fluctuations and Failures	Voltage should remain above 4.65 V.	ACT	If voltage drops below 4.75 V: Shutdown RPi to prevent data corruption.
			MSG	{“alertType”: “System Alert”, “message”: “Under-voltage detected. RPi will shut down to prevent data corruption. Check power resources.”}
sm3	High CPU Usage	Sustained usage > 90%	ACT	Terminate high-usage processes to reduce load.
			MSG	{“alertType”: “System Alert”, “message”: “High CPU usage detected. Optimization in progress.”}
sm4	Memory Leak	Free RAM consistently below 10%	ACT	Restart or fix problematic applications to free up memory.
			MSG	{“alertType”: “System Alert”, “message”: “Memory usage critical. Releasing resources.”}
sm5	Disk Space Full	Usage > 90%	ACT	Free up disk space; if usage exceeds 97%, initiate system shutdown.
			MSG	{“alertType”: “System Alert”, “message”: “Disk space critically low. Please check storage.”}
sm6	Network Latency	Latency > 100 ms	ACT	Troubleshoot network; optimize connections and hardware if necessary.
			MSG	{“alertType”: “System Alert”, “message”: “High network latency detected. Please verify network connection.”}
sm7	Peripheral Malfunction	N/A	MSG	{“alertType”: “System Alert”, “message”: “Peripheral malfunction detected. Please check device connection.”}
sm8	GPIO Pin Damage	Max 3.3 V and 16 mA per pin; total 50 mA across all pins.	MSG	{“alertType”: “System Alert”, “message”: “GPIO pin damage detected. Please check voltage and current limits.”}
sm9	SD Card Wear	N/A	MSG	{“alertType”: “System Alert”, “message”: “SD card wear detected. Consider replacing the SD card to avoid data corruption.”}
sm10	Peripheral Status	N/A	MSG	{“alertType”: “System Alert”, “message”: “Peripheral malfunction detected. Please verify device connection and compatibility.”}
sm11	Cache Memory	Cache usage > 80% of total RAM	ACT	Clear cache memory to free up resources and optimize performance when cache usage exceeds 80% of total RAM.
			MSG	{“alertType”: “System Alert”, “message”: “High cache usage detected. Clearing cache to free up memory.”}
sm12	UPS Battery Level	Battery level < 20%	ACT	Initiate safe shutdown sequence to preserve data if battery level is critically low.
			MSG	{“alertType”: “System Alert”, “message”: “UPS battery critically low. Initiating safe shutdown to prevent data loss.”}

**Table 5:** Routine Operations Scheduler.

Number	Problem	Type	Prescription	Monitoring Frequency
ros1	File System Corruption	ACT	{“action”: “Repair file system”, “description”: “Restore data integrity”}	Quarterly
ros2	Software Crashes	MSG	{“alertType”: “Prescription”, “message”: “Please update the corresponding application.”}	Biannual
ros3	Clock Drift	ACT	{“action”: “Synchronize system clock”, “description”: “Maintain accurate system time”}	Monthly
ros4	Firmware Outdated	MSG	{“alertType”: “Prescription”, “message”: “Please update your system.”}	Variable
ros5	Delete Old Logs	ACT	{“action”: “Delete old logs”, “description”: “Clear logs older than threshold.”}	Biannual
ros6	Cache and Temporary Files	ACT	{“action”: “Clear cache and delete temporary files”, “description”: “Free up space”}	Weekly
ros7	Temporary Files	MSG	{“alertType”: “Prescription”, “message”: “Temporary files require cleanup.”}	Biannual
ros8	Security Updates	MSG	{“action”: “Apply security updates”, “description”: “Maintain system security, requires user approval if biannual”}	Biannual
ros9	Backup Important Files	ACT	{“action”: “Backup files”, “description”: “Save critical files and configuration data.”}	Monthly

**Table 6:** Error Tracker Module.

Number	Problem	Variable	Type	Prescription
etm1	Overheating	Temperature (C)	MSG	{"alertType": "System Alert", "message": "Temperature is going to exceed safe limits. Please check. "}
etm2	High CPU usage	CPU usage (%), Number of Threads (int), Number of Processes (int)	MSG	{"alertType": "System Alert", "message": CPU usage is going to exceed safe limits. Please check."}
etm3	Memory Leak	Ram Memory (%), Number of Threads (int), Number of Processes (int)	MSG	{"alertType": "System Alert", "message": Ram usage is going to exceed safe limits. Please check."}
etm4	Disk Space Full	Disk Space (%)	MSG	{"alertType": "System Alert", "message": Disk usage is going to exceed safe limits. Please check."}
etm5	Cache Memory Leak	Cache Memory (%), Number of Threads (int), Number of Processes (int)	MSG	"alertType": "System Alert", "message": Cach memory usage is going to exceed safe limits. Please check."}

**Table 7:** Specifications and roles of Raspberry Pi 5 devices.

Component	Details	Notes
Operating System	Raspbian OS (Raspberry Pi OS)	Latest stable version installed
SD Card Size	32 GB	MicroSD Card Class 10, formatted for boot
Internet Connection	Wired (Ethernet)	Direct connection to router, 100 Mbps Ethernet for stable network access
Power Supply	5 V 3 A USB-C Power Adapter	Standard Raspberry Pi power adapter
Monitor	No (VNC remote access)	Remote access via VNC instead of using a physical monitor
Additional Accessories	None	No additional peripherals or accessories connected
Remote Access	Enabled (VNC)	VNC server activated for remote desktop functionality
Cooling	Yes (Active Cooling)	External fan attached for thermal regulation during operation
Backup Power	No UPS	Device operates without an uninterruptible power supply

**Table 8:** Devices and sensors in the HARN Z-Wave IoT system.

Device Name	Brand	Type/Description
Raspberry Pi 5 [26]	Raspberry	Lightweight desktop computing
Aeotec Z-Stick 7 [28]	Aeotec	Z-Wave controller for home automation
MCO Home Z-Wave CO_2_ [29]	MCO Home	CO_2_ Sensor with Z-Wave compatibility
Aeotec Heavy Duty Smart Switch Gen5 [30]	Aeotec	High-power smart switch for appliances (Z-Wave)
3-Phase Smart Meter [31]	Qubino	Energy monitoring for three-phase systems
Smart Plug Fibaro Z-Wave Plus v2 [32]	Fibaro	Smart plug with energy monitoring (Z-Wave Plus)
FIBARO Motion Sensor [33] Z-Wave Plus White	Fibaro	Motion and temperature sensor (Z-Wave Plus)
ZXT-600 AC MASTER [34]	Remotec	Z-Wave-to-IR bridge for air conditioner control
UPS HAT [35]	Waveshare	Uninterruptible Power Supply UPS HAT (B) for Raspberry Pi, 5 V Output, up to 5 A Current, Pogo Pins Connector
Internal Fan [36]	Raspberry	Raspberry Pi Active Cooler
External Fan [37]	GeeekPi	Pi Cooling Fan 30 × 30 × 7 mm DC 5 V Brushless CPU Cooling Fan

**Table 9:** Comparison of the proposed AE-LSTM model with SoA.

Confidence Value	Model	Precision	Recall	Accuracy	F1-Score
*k* = 6	AE-Dense(1-1)	0.909	0.968	0.953	0.944
	AE-Dense(2-2)	0.915	0.987	0.967	0.945
	AE-LSTM(1-1)	0.930	0.988	0.959	0.956
	AE-LSTM(2-2)	0.939	0.988	0.978	0.978
nu = 0.005 cont. = 0.002	OC-SVM	0.916	0.934	0.948	0.935
	IF	0.913	0.929	0.938	0.924
*k* = 7	AE-Dense (1-1)	0.938	0.968	0.954	0.959
	AE-Dense(2-2)	0.938	0.968	0.954	0.959
	AE-LSTM(1-1)	0.957	0.979	0.979	0.969
	AE-LSTM(2-2)	0.968	0.979	0.979	0.972
nu = 0.05 cont. = 0.003	OC-SVM	0.933	0.945	0.956	0.947
	IF	0.931	0.945	0.945	0.941
*k* = 8	AE-Dense (1-1)	0.938	0.968	0.954	0.959
	AE-Dense(2-2)	0.949	0.981	0.954	0.962
	AE-LSTM(1-1)	0.975	0.982	0.982	0.978
	**AE-LSTM (2-2)**	**0.989**	**0.999**	**0.999**	**0.990**
nu = 0.08 cont. = 0.005	OC-SVM	0.951	0.961	0.969	0.961
	IF	0.951	0.959	0.965	0.955

**Table 10:** Comparative analysis of hierarchical and non-hierarchical network performance.

Aspect	Hierarchical Network (HARN)	Non-Hierarchical Network
Number of Nodes	25 nodes (15 ARNs → 6 Neighborhoods → 3 Districts → 1 City).	25 nodes (15 ARNs communicate directly with 1 city node).
Messages per Hour	∼75 messages/h (5 messages per ARN → Neighborhood → District → City).	∼375 messages/h (15 ARNs × 25 messages/hour direct to the city node).
Latency	∼500 ms (local decisions at ARNs and neighborhood/district levels).	∼1500 ms (all decisions made centrally at city node).
Energy Consumption	∼15% of total energy allocated to communication (efficient tiered model).	∼30% of total energy allocated to communication (high communication load).
Data Traffic	∼5 MB/h (data aggregated at neighborhood and district levels).	∼25 MB/h (raw data sent directly from ARNs to the city node).
Decision-Making Speed	∼1–2 s for local-level decisions; ∼5 s for city-wide decisions.	∼8–10 s for all decisions due to bottlenecks at the city node.
Network Load	Distributed: Max node load ∼30% at city-level node.	Centralized: City node load ∼90%, risking overload.
Resilience to Failures	∼85% localized fault containment (most faults resolved at ARNs/neighborhood levels).	∼40% localized fault containment (city node must address most issues).
Scalability	Easily scalable to larger networks with tiered structure (up to 10,000 nodes).	Scalability limited to ∼100 nodes before city node becomes overwhelmed.

## Data Availability

Data are unavailable due to privacy restrictions.

## Appendix A. Coordination Algorithms and Actions

**Algorithm A1** Neighborhood Manager Coordination Algorithm.
**function** CoordinateNeighborhoodManager(NeighborhoodManager) **while** TRUE **do** ▹ Blackout Management **for all** ARN **in** Neighborhood **do** **if** ARN.UPSLevel < 10% **then** **SendCommand**(ARN, “ShutdownNonCriticalNodes”) **end if** **if** ARN.UPSLevel < 10% **and** UPSDurationRemaining < 1 **then** **SendMessage**(“Increase reporting frequency for battery status”.) **end if** **end for** ▹ Battery Status Updates **if TimeToSendBiDailyBatteryStatusUpdate() then** batteryStatuses ← **CollectARNBatteryData**() **SendToDistrictManager**(batteryStatuses) **end if** ▹ Critical Power Shortage **if CriticalPowerShortageDetected**() **then** **SendCommand**(“ReduceNonEssentialPowerUsage”) **PrioritizeCriticalServices**() **end if** ▹ Emergency Command **if EmergencyDetected**() **then** **SendCommand**(“ImmediatePowerCutToNonCriticalInfrastructure”) **end if** ▹ Routine Maintenance **if MonthlyRoutineDue() then** **SendMessage**(“Routine Maintenance Alert: Conduct diagnostics across all ARNs”.) **ConductSystemDiagnostics**() **end if** ▹ Network Traffic Monitoring **for all** ARN **in** Neighborhood **do** **if IncreasedNetworkTrafficDetected**(ARN) **then** **SendAlert**(“Potential data theft or external control detected. Investigate”.) **ReviewAccessLogs**(ARN) **end if** **end for** ▹ Peak Demand Management **if PeakDemandHoursDetected() then** **SendMessage**(“Activate load management strategies within the neighborhood”.) **end if** ▹ Monthly Tasks **if MonthlyRoutineDue() then** **SynchronizeClocks**() **SendMessage**(“Respond all: Check all ARNs for vulnerabilities”.) **end if** **Wait**(NeighborhoodUpdateInterval) ▹ Wait until next cycle **end while** **end function**

**Algorithm A2** District Manager Coordination Algorithm.
**function** CoordinateDistrictManager(DistrictManager) **while** TRUE **do** ▹ Blackout Management **for all** Neighborhood **in** District **do** **if** Neighborhood.UPSLevel < 10% **then** **SendCommand** (Neighborhood, “ShutdownNonCriticalNodes”) **end if** **if** Neighborhood.UPSLevel < 10% **and** UPSDurationRemaining < 1 **then** **SendMessage**(“Increase reporting frequency for battery status”.) **end if** **end for** ▹ Battery Status Updates **if TimeToSendBiDailyBatteryStatusUpdate() then** batteryStatuses ← **CollectNeighborhoodBatteryData**() **SendToCityPlanner**(batteryStatuses) **end if** ▹ Critical Power Shortage **if CriticalPowerShortageDetected**() **then** **SendCommand**(“ReduceNonEssentialPowerUsage”) **PrioritizeCriticalServices**() **end if** ▹ Emergency Command **if EmergencyDetected**() **then** **SendCommand**(“ImmediatePowerCutToNonCriticalInfrastructure”) **end if** ▹ Routine Maintenance **if MonthlyRoutineDue() then** **SendMessage**(“Routine Maintenance Alert: Conduct diagnostics across all neighborhood ARNs”.) **ConductSystemDiagnostics**() **end if** ▹ Network Traffic Monitoring **for all** ARN **in** District **do** **if IncreasedNetworkTrafficDetected**(ARN) **then** **SendAlert**(“Potential data theft or external control detected. Investigate”.) **ReviewAccessLogs**(ARN) **end if** **end for** ▹ Peak Demand Management **if PeakDemandHoursDetected() then** **for all** Neighborhood **in** District **do** **SendMessage**(“Activate neighborhood-level load management strategies”.) **end for** **end if** ▹ Monthly Tasks **if MonthlyRoutineDue() then** **SynchronizeClocks**() **SendMessage**(“Respond all: Check all systems for vulnerabilities”.) **end if** **Wait**(DistrictUpdateInterval) ▹ Wait until next cycle **end while** **end function**

**Algorithm A3** City Planner Coordination Algorithm.
**function** CoordinateCityPlanner(CityPlanner) **while** TRUE **do** ▹ Blackout Management **for all** District **in** City **do** **if** District.UPSLevel < 10% **then** **SendCommand**(District, “ShutdownNonCriticalNodes”) **end if** **if** District.UPSLevel < 10% **and** UPSDurationRemaining < 1 **then** **SendMessage**(“Increase reporting frequency for battery status”.) **end if** **end for** ▹ Battery Status Updates **if TimeToSendBiDailyBatteryStatusUpdate() then** batteryStatuses ← **CollectDistrictBatteryData**() **SendToCityPlannerManager**(batteryStatuses) **end if** ▹ Critical Power Shortage **if CriticalPowerShortageDetected**(DistrictX) **then** **SendCommand**(DistrictX, “ReduceNonEssentialPowerUsage”) **PrioritizeCriticalServices**(DistrictX) **end if** ▹ Emergency Command **if EmergencyDetected**(DistrictX) **then** **SendCommand**(DistrictX, “ImmediatePowerCutToNonCriticalInfrastructure”) **end if** ▹ Routine Maintenance **if MonthlyRoutineDue() then** **SendMessage**(“Routine Maintenance Alert: Conduct diagnostics across all district ARNs”.) **ConductSystemDiagnostics**() **end if** ▹ Network Traffic Monitoring **for all** ARN **in** City **do** **if IncreasedNetworkTrafficDetected**(ARN) **then** **SendAlert**(“Potential data theft or external control detected. Investigate”.) **ReviewAccessLogs**(ARN) **end if** **end for** ▹ Peak Demand Management **if PeakDemandHoursDetected() then** **for all** District **in** City **do** **SendMessage**(“Activate district-level load management strategies to prevent grid strain”.) **end for** **end if** ▹ Monthly Tasks **if MonthlyRoutineDue() then** **SynchronizeClocks**() **SendMessage**(“Respond all: Check all systems for vulnerabilities”.) **end if** **Wait**(CityUpdateInterval) ▹ Wait until next cycle **end while** **end function**

**Table A1:** City Planner Actions.

Category	Functions	Description	Frequency
Power Management & Distribution	Blackout Message	Monitor UPS status to identify affected areas. If an ARN battery drops below 30%, prompt data upload; below 10%, issue a shutdown alert. Battery checks occur every 10 min.	As needed during blackout
	City Planner Shutdown due to Low Battery	When city planner’s battery level falls below 10%, initiate shutdown procedures to conserve power. District Manager is informed.	As needed, if battery <10%
	Critical Power Shortage Alert	Instructs specified district(s) to reduce non-essential power usage and prioritize critical services.	As needed during shortage
	Load Balancing Strategy	Issues city-wide load management strategies, distributing power to maintain grid stability, especially during peak hours.	During peak hours
	Overload Warning	Alerts neighborhoods nearing consumption thresholds, prompting load reduction to prevent outages.	Hourly
Emergency Response & Alerts	Emergency Command	Initiates immediate power cuts to non-critical infrastructure in specified district(s) in response to emergencies.	As needed during emergency
	Backup and Fire News	Enables sending the data to the cloud, and sends a notification message	As needed
Routine Maintenance & Diagnostics	Routine Maintenance Alert	Diagnostic check across all district ARNs to identify vulnerabilities or inefficiencies.	Monthly
	Routine System Checks	Clock synchronization, city-wide response checks for all nodes.	Monthly
	Energy Efficiency Report	Compiles usage data from all nodes to evaluate city-wide energy efficiency and optimize future resource allocation.	Quarterly
Security & Data Monitoring	Increased Network Traffic	Monitors for unusual data flow in ARNs, potentially indicating unauthorized access or external control connections.	As needed
	Security Alert	Periodically reviews access logs to detect unauthorized patterns, especially during peak network activities.	Weekly

**Table A2:** District Manager Actions.

Category	Functions	Description	Frequency
Power Management & Distribution	Blackout Message	Monitor UPS status to identify affected areas within the district. If an ARN battery drops below 30%, prompt data upload; below 10%, issue a shutdown alert. Battery checks occur every 10 min.	As needed during blackout
	District Manager Shutdown due to Low Battery	Initiate shutdown procedures if the district’s primary node battery falls below 10% to conserve power. District manager is informed and can prepare alternative solutions.	As needed, if battery <10%
	Critical Power Shortage Alert	Instruct neighborhoods within the district to reduce non-essential power usage and prioritize critical services.	As needed during shortage
	Load Balancing Strategy	Implements district-wide load distribution strategies to maintain stability, especially during peak hours.	During peak hours
	Overload Warning	Alerts neighborhoods within the district nearing consumption thresholds, prompting load reduction to prevent outages.	Hourly
Emergency Response & Alerts	Emergency Command	Initiates immediate power cuts to non-critical infrastructure within the district in response to emergencies.	As needed during emergency
	Backup and Fire News	Ensures data are sent to the cloud, and sends a notification to appropriate district-level contacts in case of fire or backup activation.	As needed
Routine Maintenance & Diagnostics	Routine Maintenance Alert	Conducts diagnostic checks across all ARNs within the district to identify vulnerabilities or inefficiencies.	Monthly
	Routine System Checks	Synchronizes clocks and checks response readiness for all nodes within the district.	Monthly
	Energy Efficiency Report	Aggregates and analyzes usage data from all district nodes to evaluate energy efficiency and optimize allocation.	Quarterly
Security & Data Monitoring	Increased Network Traffic	Monitors unusual data flow within the district’s ARNs, potentially indicating unauthorized access or control issues.	As needed
	Security Alert	Regularly reviews access logs within the district to detect unauthorized access, especially during peak activities.	Weekly

**Table A3:** Neighborhood Manager Actions.

Category	Functions	Description	Frequency
Power Management & Distribution	Blackout Message	Monitor UPS status to identify affected areas within the neighborhood. If an ARN battery drops below 30%, prompt data upload; below 10%, issue a shutdown alert. Battery checks occur every 10 min.	As needed during blackout
	Neighborhood Manager Shutdown Due to Low Battery	Initiate shutdown procedures if the neighborhood’s primary node battery falls below 10% to conserve power. Neighborhood Manager is informed to prepare alternative solutions.	As needed, if battery <10%
	Critical Power Shortage Alert	Direct homes and facilities within the neighborhood to reduce non-essential power usage and prioritize critical services.	As needed during shortage
	Load Balancing Strategy	Implements load distribution strategies within the neighborhood to maintain stability, especially during peak hours.	During peak hours
	Overload Warning	Alerts homes and facilities within the neighborhood nearing consumption thresholds, prompting load reduction to prevent outages.	Hourly
Emergency Response & Alerts	Emergency Command	Initiates immediate power cuts to non-critical infrastructure within the neighborhood in response to emergencies.	As needed during emergency
	Backup and Fire News	Ensures data are sent to the cloud and sends a notification to neighborhood-level contacts in case of fire or backup activation.	As needed
Routine Maintenance & Diagnostics	Routine Maintenance Alert	Conducts diagnostic checks across all ARNs within the neighborhood to identify vulnerabilities or inefficiencies.	Monthly
	Routine System Checks	Synchronizes clocks and checks response readiness for all nodes within the neighborhood.	Monthly
	Energy Efficiency Report	Aggregates and analyzes usage data from all neighborhood nodes to evaluate energy efficiency and optimize allocation.	Quarterly
Security & Data Monitoring	Increased Network Traffic	Monitors unusual data flow within the neighborhood’s ARNs, potentially indicating unauthorized access or control issues.	As needed
	Security Alert	Regularly reviews access logs within the neighborhood to detect unauthorized access, especially during peak activities.	Weekly
